# Splint Duration and Not the Mode of Anesthesia Is the Main Factor Influencing Avascular Necrosis After Closed Reduction for Developmental Dysplasia of the Hip in Kosovo

**DOI:** 10.3389/fped.2022.850605

**Published:** 2022-04-26

**Authors:** Sabit Sllamniku, Besiana P. Beqo, Islam Krasniqi, Azem Tërshana, Ardiana Murtezani, Franz Quehenberger, Emir Q. Haxhija

**Affiliations:** ^1^Orthopedic Department, University Clinical Center of Kosovo, Prishtina, Republic of Kosovo; ^2^Department of Postgraduate Medical Education, Global Clinical Scholars Research Program, Harvard Medical School, Boston, MA, United States; ^3^Department of Pediatric and Adolescent Surgery, Medical University of Graz, Graz, Austria; ^4^Department of Anesthesiology, University Clinical Center of Kosovo, Prishtina, Republic of Kosovo; ^5^Department of Pediatrics, Medical Faculty of Prishtina University, Prishtina, Republic of Kosovo; ^6^Physical Medicine and Rehabilitation Department, University Clinical Center of Kosovo, Prishtina, Republic of Kosovo; ^7^Institute of Medical Informatics, Statistics and Documentation, University of Graz, Graz, Austria

**Keywords:** developmental dyspalsia of the hip, analgesia and sedation, general anesthesia, splint duration, closed reduction

## Abstract

The aim of this study was to determine whether the use of analgesia and sedation (AS) as opposed to general anesthesia (GA) for closed reduction and spica casting of children with severe developmental dysplasia of the hip (DDH) influenced the long-term incidence of avascular necrosis (AVN). In a prospective, randomized, single-blinded clinical trial we investigated 100 pediatric patients with DDH type IIIa, IIIb, and IV (according to Graf classification), who were randomly assigned into the group receiving AS, and the group receiving GA. Baseline demographics, splint duration, and type of DDH were carefully assessed. The presence of AVN was assessed at the follow-up visits at 1 and 7 years after the end of treatment. The AS-group consisted of 50 patients (46 girls) with 76 hips affected (*n* = 11/Type-IIIa, *n* = 32/Type-IIIb, and *n* = 33/Type-IV). The GA-group consisted also of 50 patients (44 girls) with 78 hips involved (*n* = 15/Type-IIIa, *n* = 34/Type-IIIb, and *n* = 29/Type-IV). At 7-years follow-up, AVN was diagnosed in 9 of 154 hips (5.8%), 5 hips in the AS-group and 4 hips in the GA group. The logistic regression model showed no significant difference in AVN incidence between the AS and GA groups at 7-years follow-up (*p* = 0.27). The multivariate regression analysis showed that neither the type of DDH nor the age at diagnosis influenced the incidence of AVN (*p* = 0.48 and *p* = 0.28, respectively). Splint duration was identified as the only significant factor for the long-term incidence of AVN in the treatment of severe DDH. For every month of longer splint duration, the odds of AVN at 7-years follow-up increased by a factor of 3.81 (95%*CI*: 1.35–13.73, *p* = 0.02). Closed reduction and spica casting of children with severe DDH under AS can be considered a feasible alternative to management under GA. All efforts must be made to diagnose patients with DDH as early as possible and shorten the duration of splint treatment to prevent the development of AVN. Level of Evidence. Level II-1.

## Introduction

Developmental dysplasia of the hips (DDH) remains a prominent pathology among children in Kosovo (1). Amid 31,002 live births in our country from 2011 to 2012, the overall rate of those presented with hip dislocation was 0.25% (2). During this period, the reported incidence rate of newborns treated with closed reduction (percutaneous traction, spica casting in human position, and dynamic splinting with Tübingen hip–flexion orthosis) was 0.18%, and the rate of cases treated with surgery was 0.07%. The closed reduction and spica casting are commonly performed procedures in our department to obtain concentric hip reduction and allow for acetabular and femoral remodeling with the lowest avascular necrosis (AVN) rate (3, 4). Bracing and splinting are shown to be the first line of DDH management with a low rate of AVN reported (5, 6).

Graf’s classification of hip dysplasia by ultrasound is a valuable tool for early recognition of all degrees of DDH, but due to lack of appropriate infrastructure, despite high incidence rate of DDH in our country, unfortunately, Kosovo still does not have a national screening program for DDH.

The incidence rate of AVN after closed reduction management of DDH ranges between 4 and 60% (3, 7). The main cause of AVN after closed reduction is iatrogenic and the main risk for developing AVN is late presenting children regardless of hip dysplasia classification (1). Previous reports have shown that early diagnosis of DDH, preliminary traction, gentle reduction under the use of general anesthesia (GA), adductor tenotomy, and avoidance of an extreme position of immobilization have helped decrease the incidence of AVN (3, 4, 7–10).

Although GA has been reported to be among the protective factors against the development of AVN (7, 8, 11, 12) in our institution closed reduction and spica casting was traditionally performed under analgesia and sedation (AS) due to limited resources in the operating theater. So far, the relationship between the type of used anesthesia and AVN is not yet explored.

The purpose of this study was to investigate whether there was any difference on the long-term AVN incidence depending on the type of anesthesia used during closed reduction treatment of DDH. We hypothesized that the incidence of AVN will be lower in children treated under GA as compared to those treated under AS.

## Materials and Methods

### Study Design

The study is a prospective, randomized, single-blinded clinical trial with an 8-years assessment period and two-point in time follow-up visits. The first follow-up assessment is done in all trial patients 1 year after the end of the treatment. Only patients diagnosed with AVN during the first visit were subjected to a second follow-up assessment 7 years after the end of treatment. The interventional part of the study was conducted from January 2011 through October 2012. Final follow-up visits were conducted from June 2019 to December 2020.

The primary objective was to investigate whether the use of AS or GA during closed reduction management of DDH has any significant effect on subsequent AVN development. The secondary objectives were to investigate the role of patients’ age at diagnosis, gender, the number of affected hips, the affected side (right/left), the degree of initial hip dysplasia, and the duration of splinting on AVN development afterward. The only difference in the course of the treatment of these patients was the mode of anesthesia for closed reduction.

Online randomization^1^ for 200 patients was used to generate the randomization plan for two groups in a block of two patients to control for equal numbers between the groups. Subject recruitment was terminated after 100 patients were included in the trial.

The trial protocol was approved by the University Ethical Committee for Medical and Health Research of our country with approval number 02-2010. Signed and dated written informed consent of the parent (or the person having parental authority in the family) is obtained according to the 1964 Declaration of Helsinki.

**FIGURE 1:**
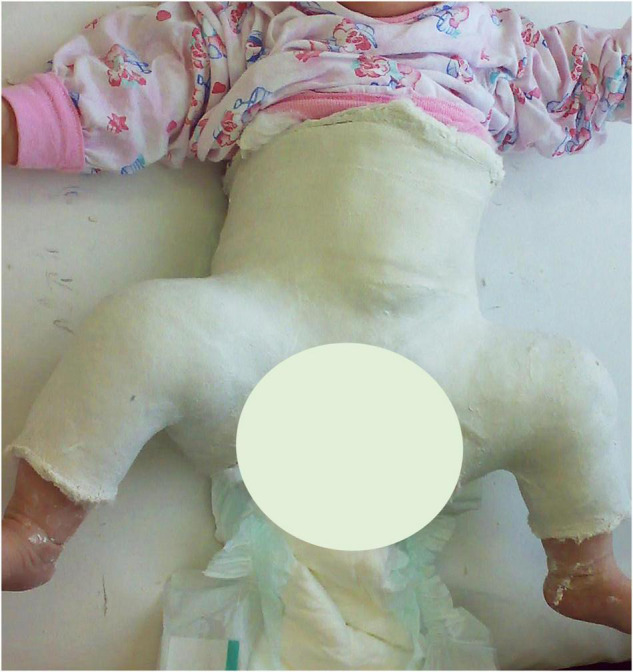
A 4.5-months-old baby-girl with left hip dislocation after closed reduction and spica casting.

At our institution, the standard treatment for children diagnosed with DDH type IIIa/IIIb/IV consists of inpatient percutaneous Bryan’s traction with approximately 10% of child body weight as weighted traction for 2 weeks, followed by closed reduction with arthrography, adductor tenotomy when a narrow safe zone is presented, and spica casting in human position (90–100 degrees flexion and 45–60 degrees abduction) for a period of 4 weeks (Figure 1). None of the patients wear Tübingen splint before casting. Further treatment is continued in the outpatient setting. Tübingen hip–flexion orthosis is used after removing the cast until we consider that the femoral head position is normal and stable throughout the ultrasound examination (13). Tübingen splint is removed only during clinical and US examination. We use Tübingen splint for every patient independently from age and body weight. Clinical and ultrasound checkups were conducted at intervals of 4 weeks. After 6 months of age, X-ray checkups were necessary.

### Study Population

**FIGURE 2:**
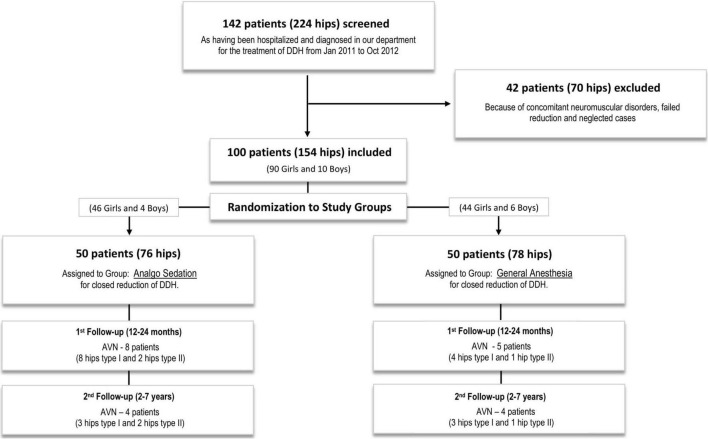
The CONSORT flowchart shows the selection and randomization of the study subjects in two different groups of the clinical trial.

All consecutive patients diagnosed with DDH type IIIa/IIIb/IV (according to Graf’s classification) (14), during the study period were initially randomized into the study. Only patients with successful closed reduction and successful transition from spica casting to Tübingen splint were included into the study. Exclusion from the study followed 42 patients with 70 abnormal hips (28 bilateral, 8 left and 6 right) who all needed surgical intervention by Salter’s and/or Pemberton’s osteotomies combined or not with femoral osteotomies (15). Teratologic hip dislocation is seen in two of the patients, 2 patients failed closed reduction in both groups by one, and 38 patients were considered neglected cases, which means they were not diagnosed before walking age. After these exclusions, 100 patients (90 girls and 10 boys) with 154 dislocated hips (54 bilateral, 28 left, and 18 right) were included in the present study. Figure 2 describes the sample selection. Percutaneous adductor muscle tenotomy was performed in 12 patients, 5 in the AS group, and 7 in the GA group.

Perioperative management of all the patients consisted of administering midazolam 0.5 mg/kg BW orally with apple juice and paracetamol 15 mg/kg BW supp. rectally 30 min before the intervention, followed by transportation of the patients into the operation theater in their beds with legs under traction.

Patients were randomly allocated into two parallel groups: the AS group initially received iv fentanyl 1 mcg/kg BW and subsequently 1 mcg/kg as required (16, 17). The GA group received fentanyl 2–6 mcg/kg BW iv and later a maintenance dose of 0.5–2 mcg/kg BW (18). In addition, a bolus dose of iv propofol 2–3 mg/kg BW was administered, and for maintenance of anesthesia, 5–15 mg/kg/hour. Finally, an inhalational induction dose of sevoflurane 5% and for maintenance 0.5–2% was given (19). Airway management was performed by Ambu mask in the AS group and by laryngeal mask in the GA group. Postoperative management consisted of analgesia with paracetamol given as suppository 15 mg/kg BW t.i.d (20). Patients were discharged home with a spica cast on the first post-intervention day after re-evaluation was done by the orthopedic surgeon and the anesthesiologist.

### Data Collection

Collected data for every study subject consisted of baseline demographics, clinical, and imaging details, for each included hip from the time of initial consultation to the most recent follow-up. At the baseline visit, clinical examination and ultrasound (US) was performed by the orthopedic surgeon to establish a full spectrum of DDH severity according to Graf sub-types (14). In all children US is used for evaluation and clinical decision-making. However, US is limited to being less accurate with continued growth and ossification of the femoral head in the older infant (21). To this extent, anteroposterior pelvic radiographs were used in addition to US for assessing hips of the patients older than 6 months for the position of the hip reduction, the possibility of failure of reduction, and the radiological parameters that indicate the development of the osteonecrosis of the femoral head (14, 22). The radiographs were evaluated by both, the radiologists and the orthopedic surgeons in charge of the patient.

The first follow-up examination was performed for all patients 1 year after the end of treatment. Patients diagnosed with AVN at the 1st follow-up visit were referred for a second re-evaluation and follow-up was 7 years after the end of treatment. Both assessments were made by anteroposterior pelvic radiographs. Every patient’s parents were reminded by phone call, specifically before the scheduled follow-up visit, to ensure full compliance. No patients were lost to follow-up.

**FIGURE 3:**
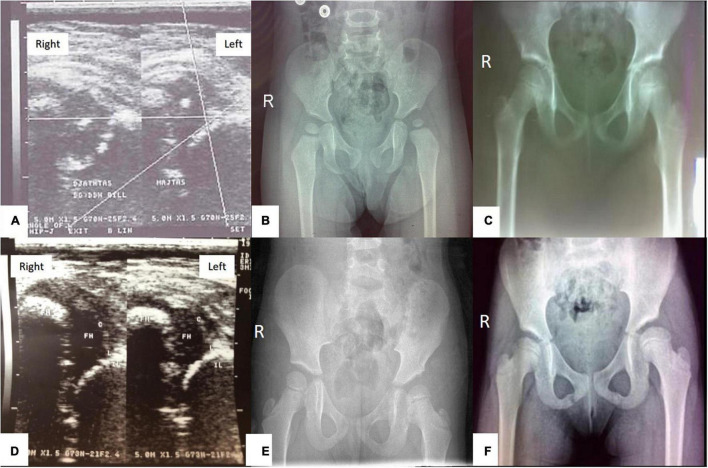
A baby girl with left hip dislocation undergoing closed reduction under analgesia and sedation **(A–C),** and a baby girl with bilateral hip dislocation undergoing closed reduction under general anesthesia **(D–F)**: **(A)** Ultrasound examination of the hips at the age of 4.9 months showing left hip dislocation type IIIb according to Graf’s classification; **(B)** radiography of the hips at the age of 20 months, showing AVN type I of the left femoral head according to Ogden and Bucholz classification- hypoplastic epiphysis of the proximal femur; **(C)** radiography of the hips at the age of 7.9 years showing complete improvement of ossification of the left femoral head. **(D)** Ultrasound examination of the hips at the age of 3.9 months showing bilateral hip dislocation type IV according to Graf’s classification; **(E)** Radiography of the hips at the age of 2.4 years showing AVN type II of the left femoral head according to Ogden and Bucholz classification with overgrowth of greater trochanter into valgus, lateral metaphysis shows evidence of injury; **(F)** radiography of the hips at the age of 7.6 years showing AVN type II of the left femoral head according to Ogden and Bucholz classification – overgrowth of greater trochanter into valgus.

The presence of AVN in the follow-up radiographic evaluation was assessed by the criteria of Bucholz and Ogden’s classification (22) (Figure 3). Still, for the purpose of this manuscript and for statistical analysis each hip was only classified and calculated as “1/YES” vs. “0/NO” for radiographic evidence of AVN.

### Statistical Analysis and Study Variables

The exact Wilcoxon test was used to analyze the difference between the two clinical trial groups for continuous features. Logistic regression analysis was performed to find a relationship between the independent (predictor) and dependent (target) variables in the closed reduction of DDH. Our study’s outcomes (dependent variables) were the frequency of AVN 1 year and 7 years after the end of treatment. The investigated risk factors (independent variables) were the type of anesthesia used during closed reduction (AS vs. GA), the DDH stages IIIa/IIIb/IV (coded numerically as 1/2/3, respectively), the age at diagnosis in months, gender, the number of affected hips, the affected side (right/left), and splint duration in months. Univariate models were calculated separately for each risk factor. Multivariate models estimated a common linear predictor from the risk factors in the model. The dependence of splint duration from anesthesia, DDH stages, gender, the number of affected hips, the affected side (right/left), and age at diagnosis was similarly investigated by linear regression analysis.

Statistical significance was set a *p*-value <0.05. Categorical variables are reported as percentage and frequency. Continuous variables are presented as a measure of central tendency (mean or median) and spread (*SD* or range). Confidence intervals (*CI*) are calculated at the 95% level.

Statistical computing and graphics are performed using R.4.0.2 software.

## Results

Patient demographic data are described in Table 1. A total of 154 hips were treated in 100 children. There was no statistically significant difference in the incidence of bilateral DDH or the affected hip side between the groups. Also, there was no statistical difference in the type of DDH between the two study groups (*p* = 0.35, exact Wilcoxon test).

**TABLE 1:** Demographic characteristics of patients.

	AS	GA	Total
N of patients	50	50	100
Age at diagnosis in months (median and range)	3.6 (2.3–8.6)	3.1 (1.6–8.7)	3.3 (1.6–8.7)
Girls N (%)	46 (92%)	44 (88%)	90 (90%)
Boys N (%)	4 (8%)	6 (12%)	10 (10%)
Bilateral DDH N (%)	26 (52%)	28 (56%)	54 (54%)
Unilateral DDH N (%)	24 (48%)	22 (44%)	46 (46%)
Total number of hips with DDH	76	78	154
Left hip (N)	40	42	82
Right hip (N)	36	36	72
**Type of DDH N (%)**			
IIIa	11 (15%)	15 (19%)	26 (17%)
IIIb	32 (42%)	34 (44%)	66 (43%)
IV	33 (43%)	29 (37%)	62 (40%)

*N, number; AS, analgesia and sedation; GA, general anesthesia; DDH, developmental dysplasia of the hip.*

There was an overall strong female predominance (90%) without significant differences between the groups. Treatment was done in 138 hips in female patients and 16 hips in male patients.

The age at diagnosis ranged from 1.6 to 8.7 months, with a mean of 3.7 (± 1.4) months and a median of 3.3 months. Children in the GA group were significantly younger than children in the AS group (*p* = 0.016, exact Wilcoxon test).

**FIGURE 4:**
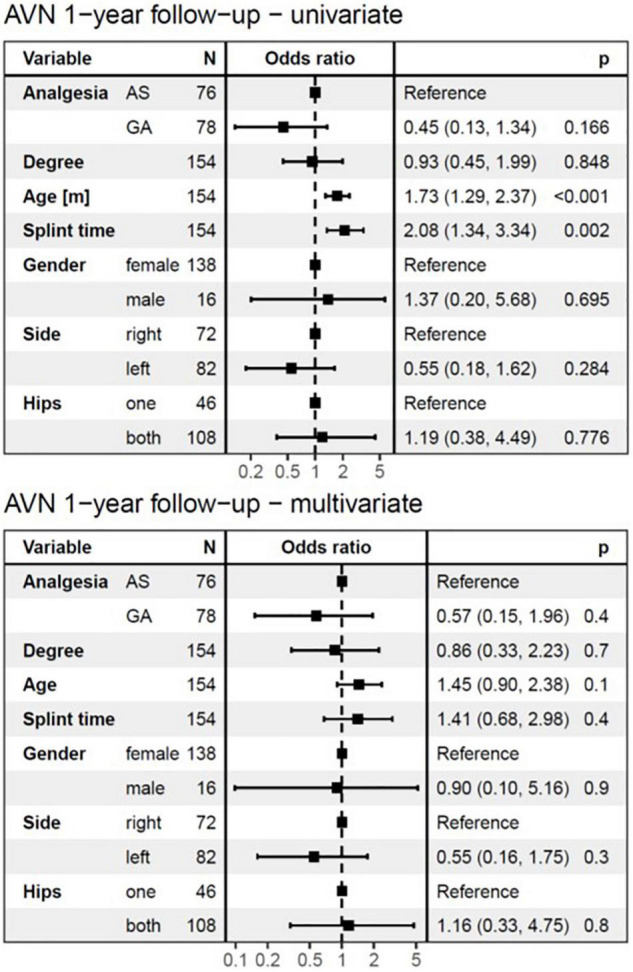
The logistic regression model at 1-year follow-up. The univariate analysis showed no significant difference in the development of AVN between AS and GA groups and identified age at diagnosis and splint time as substantial risk factors. In the multivariate analysis, none of the independent variables showed a significant influence on the development of AVN at 1-year follow-up. AVN, avascular necrosis; AS, analgesia and sedation; GA, general anesthesia; N, number of affected hips.

Avascular necrosis was diagnosed in 15/154 hips (9.7%), 1 year after completing treatment. At 7 years follow-up AVN was still present in 9/15 hips (5.8%) (Figure 4 and Table 2).

**TABLE 2:** Outcome characteristics of patients.

	AS	GA	Total
N of patients	50	50	100
AVN at 1 year (N of patients)	8 (16%)	5 (10%)	13 (13%)
AVN at 7 years (N of patients)	4 (8%)	4 (8%)	8 (8%)
N of hips in groups	76	78	154
AVN at 1 year (N of hips)	10 (13%)	5 (6%)	15 (10%)
AVN at 7 years (N of hips)	5 (7%)	4 (5%)	9 (6%)
AVN Left hip at 1/7 years (N)	5/2	1/1	6/3
AVN Right hip at 1/7 years (N)	5/3	4/3	9/3
**Type of DDH in AVN hips at 1/7 years (N)**			
IIIa	3/1	1/1	4/2
IIIb	4/1	0/0	4/1
IV	3/3	4/3	7/6

*N, number; AS, analgesia and sedation; GA, general anesthesia; AVN, avascular necrosis; DDH, developmental dysplasia of the hip.*

**FIGURE 5:**
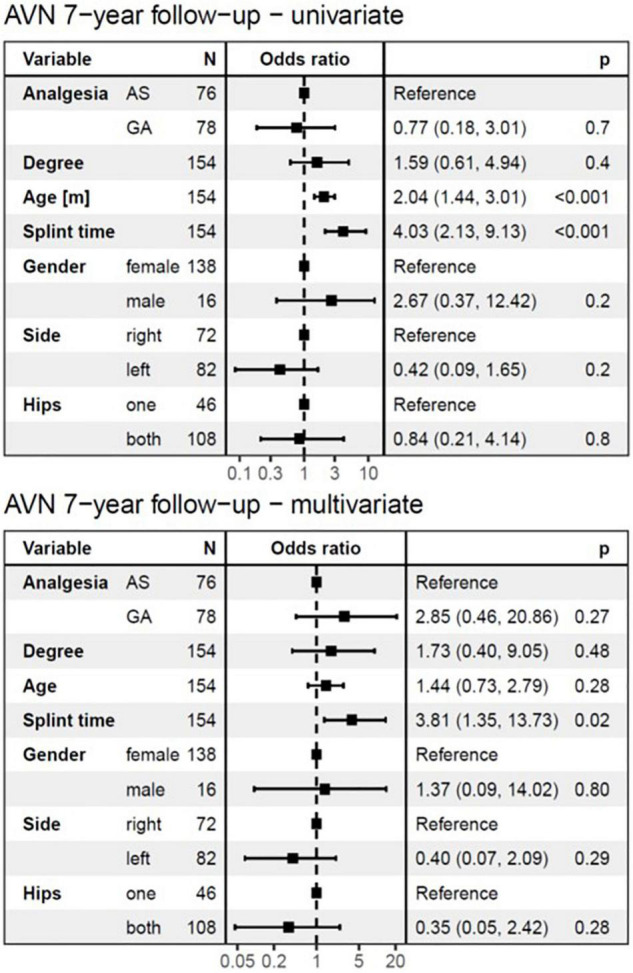
The logistic regression model at 7-years follow-up. The univariate analysis showed no significant difference in the development of AVN between AS and GA groups and identified age at diagnosis and splint time as substantial risk factors. In the multivariate analysis, only splint duration showed a significant influence on the development of AVN at a 7-years follow-up. For every month of longer splint duration for the treatment of DDH, the odds of AVN at 7-years follow-up increased by a factor of 4.03 (95%*CI*: 2.13–9.13, *p* < 0.001) in univariate analysis, and 3.81 (95%CI: 1.35–13.73, *p* = 0.02) in multivariate analysis. AVN, avascular necrosis; AS, analgesia and sedation; GA, general anesthesia; N, number of affected hips.

The logistic regression model (Figures 4, 5) for AVN showed no significant difference in the development of AVN between AS and GA groups. Therefore, the mode of anesthesia did not affect the occurrence of AVN. Both the univariate and multivariate analyses revealed that the type (degree) of DDH was not a significant risk factor for AVN development, which was true for both follow-up periods.

The univariate logistic regression model identified age at diagnosis as a significant risk factor for AVN development (*p* < 0.001), for both follow-up periods (Figures 4, 5). However, with splint duration in the multivariate analysis, age at diagnosis became an insignificant factor (*p* = 0.1).

Splint duration was identified as the only significant variable, which affected AVN at 7-years follow-up in multivariate analysis (*p* = 0.02). For every 4-week prolongation of splint duration, the risk of AVN at 1-year follow-up increased by a factor of 2.08 (95%*CI*: 1.34–3.34, *p* < 0.002) in univariate analysis and 1.41 (95%*CI*: 0.68–2.98, *p* = 0.4, not significant) in multivariate analysis (Figures 4, 5). For every 4-week of splint duration prolongation for the treatment of DDH, the odds of AVN at 7-years follow-up increased by a factor of 4.03 (95%*CI*: 2.13–9.13, *p* < 0.001) in univariate analysis, and 3.81 (95%*CI*: 1.35–13.73, *p* = 0.02) in multivariate analysis.

**FIGURE 6:**
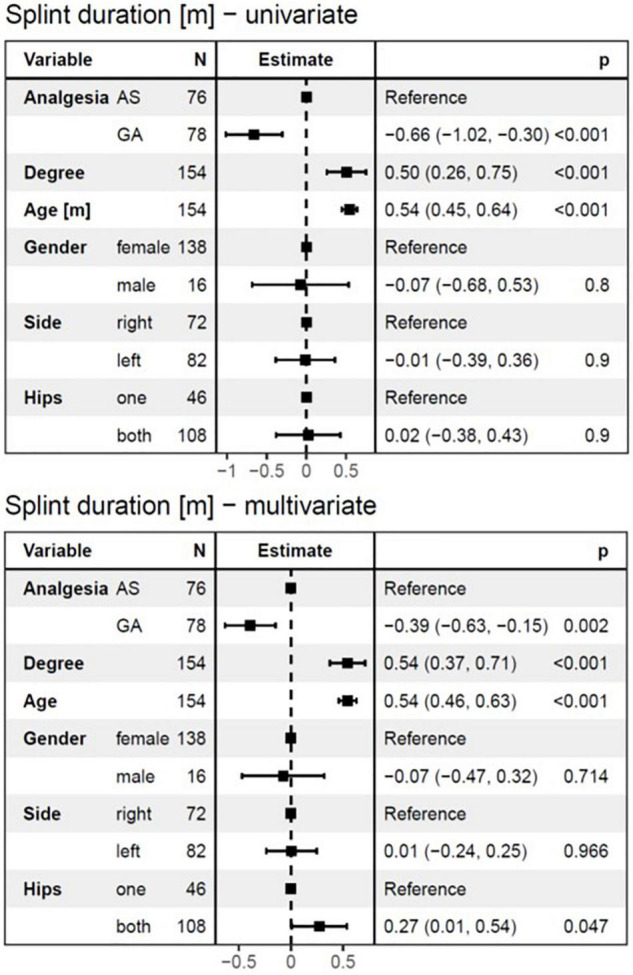
The linear regression model for splint duration as a dependent variable. Both univariate and multivariate analyses showed a highly significant influence of age at diagnosis and the degree of DDH on the splint duration. Furthermore, splint duration was –0.66 months (95%*CI*: –1.03 to –0.3, *p* < 0.001) shorter in the GA group than in the AS group in the univariate analysis, and –0.39 months (95%CI: –0.63 to –0.15, *p* = 0.002) in multivariate analysis. Interestingly, affection of both hips by DDH led to a 0.27 months longer splint duration (95%CI:0.01–0.54, *p* < 0.05) in the multivariate analysis. AS, analgesia and sedation; GA, general anesthesia; N, number of affected hips.

The overall duration of splint treatment ranged from 2 to 7.2 months with a mean of 4.1 (± 1.1) months and a median of 4.1 months. The results of univariate and multivariate linear regression analysis for splint duration are given in Figure 6. Splint duration was −0.66 months (95%*CI*: −1.03 to −0.3, *p* < 0.001) shorter in the GA group than in the AS group. The effect was −0.39 months (95%*CI*: −0.63 to −0.15, *p* = 0.002) after adjustment for independent variables in multivariate analysis. If the degree of dysplasia is higher by one stage, the splint duration is increased by 0.5 months in univariate analysis and 0.543 months in multivariate analysis (*p* < 0.001, respectively). For every month of age that the DDH treatment is delayed, splint duration is expected to increase by 0.54 months in both, univariate and multivariate analysis (*p* < 0.001, respectively). Finally, affection of both hips by DDH led to an increase of splint duration by 0.27 months (95%*CI*: 0.01–0.54, *p* < 0.05) in the multivariate analysis.

## Discussion

The present study shows that closed reduction and spica casting under AS or GA in children with DDH leads to similar outcomes concerning the incidence of AVN at 7-years follow-up. In addition, splint duration was identified as a major significant risk factor for AVN at a 7-years follow-up.

Analgesia and sedation for closed reduction and spica casting in children with DDH has not been previously reported in the literature. Because of the limited resources in our country, this technique is a common approach in our department, which was one of the preconditions to perform this study. For AS, we used a combination of oral midazolam and intravenous fentanyl as well as readily available tools for the administration of supplemental oxygen, ventilation support, and resuscitation (17, 23).

A large body of preclinical research has demonstrated that general anesthetics and sedatives, administered during critical periods of development, can have neurotoxic effects on the developing brain and neurobehavioral, neurocognitive impairments. Neurotoxic changes have been proven in experimental animals. However, the ability of these agents to induce neurotoxicity in pediatric patients has yet to be confirmed. Findings in human beings aren’t conclusive enough to illustrate the neurotoxic effects in the brains of children under 3 years old. During exposure to AS and GA the type of anesthetics, dosage, and exposure to the change. Thus, in GA the use of larger amounts and prolonged exposure to anesthetics increase the possibility of neurodevelopmental effects in the brain. Prospective studies are needed to determine definitive changes in developing brains (24–29).

Developmental dysplasia of the hip is a common pathology in children in our country. One of the reasons is traditional straightening and tight swaddling of a baby’s legs, especially in females. The other reason lies in the limited possibilities for ultrasound hip screening. Treatment of severe types of DDH is still a tremendous clinical burden for our department, which considers both children with hips that are manageable with closed reduction and children with neglected hips and primary need for surgical intervention by osteotomies.

The primary long-term complication of closed reduction and spica casting of developmentally dislocated hips is AVN of the femoral head due to diminished blood supply to the femoral epiphysis (3). In our study the incidence of AVN was 5.8%, which matches the literature data (3, 4).

The bilateral dislocation was diagnosed in 54% of our study patients, which is higher than the published data in the literature (1, 30). This is probably due to swaddling of the lower extremities fully extended and wrapped together. Newborn hips are at risk of developing dysplasia and then dislocation after swaddling.

To further evaluate variables that could influence the occurrence of AVN in the long-term outcome of our patients, we have analyzed independent variables, such as the age of children at diagnosis of DDH, the degree of DDH at diagnosis, gender, the number of affected hips, the affected side (right/left), and the splint duration in a logistic regression analysis model. This multivariate model showed that only splint duration had a significant influence on the incidence of AVN at 7-years follow-up, i.e., the longer the splint duration, the higher the incidence of AVN at 7-years follow-up. The relationship between splint duration and AVN has been reported previously in the literature (1, 3, 4, 31). The splint duration over 4 weeks in older children has been defined by some authors as a risk factor for the development of AVN (3, 4). It has been speculated that by prolonging the splint duration, the chance to help the child to save the hip by open surgery might be missed (3, 4). Elseways, open reduction of DDH has been reported to be associated with twice as a higher risk of developing AVN of the femoral head as compared to closed reduction (3).

We examined the splint duration as a dependent variable by using the linear regression model. Splint duration lasted significantly longer in older children, in children with a higher degree of dysplasia, in children with both hips affected, and in children that were treated under AS. These variables seem to indirectly affect the incidence of AVN over prolonging splint duration.

Our study’s limitation is that the randomization process did not consider the age of the included children. Unfortunately, the children randomized in the GA group were significantly younger than the children undergoing AS for closed reduction and spica casting. However, the logistic regression model corrects this difference. The older age of children at diagnosis and the degree of dysplasia remain high-risk factors for AVN development, although statistical significance was not reached in our study. Previous studies showed that AVN was more likely to occur in patients with a higher degree of initial dislocation and older age at diagnosis (7, 31). Furthermore, our findings could have been influenced by the initial management of our patients.

## Conclusion

Closed reduction and spica casting of children with DDH under AS can be a feasible alternative to management under GA. All efforts must be put into early diagnosis of DDH and early treatment starting, as it reduces the need for long splint duration, which may be the leading risk factor for AVN development at 7-years follow-up. We believe that by introduction of a mandatory ultrasonographic screening program according to Graf’s classification, also in Kosovo, the incidence of high-degree DDH will decrease.

## Data Availability Statement

The raw data supporting the conclusions of this article will be made available by the authors, without undue reservation.

## Ethics Statement

This interventional study was conducted at the Orthopedic Department of Kosovo University’s Clinical Center in Kosovo. The trial protocol was reviewed and approved by Kosovo’s University Ethical Committee for Medical and Health Research. Signed and dated written informed consent of the parent (or the person having parental authority in the family) is obtained according to the 1964 Declaration of Helsinki. Written informed consent to participate in this study was provided by the participants’ legal guardian/next of kin.

## Author Contributions

SS and BB contributed in design, data acquisition, data analysis, data interpretation, and drafting of the manuscript. IK, AT, and AM contributed in data acquisition, and critical revision of the manuscript. FQ contributed in statistical processing of data and their interpretation. EH contributed in interpretation of data, drafting and critical revision of the manuscript. All authors provide final approval of the version to be submitted.

## Conflict of Interest

The authors declare that the research was conducted in the absence of any commercial or financial relationships that could be construed as a potential conflict of interest.

## Publisher’s Note

All claims expressed in this article are solely those of the authors and do not necessarily represent those of their affiliated organizations, or those of the publisher, the editors and the reviewers. Any product that may be evaluated in this article, or claim that may be made by its manufacturer, is not guaranteed or endorsed by the publisher.

## Footnotes

1
http://www.graphpad.com/quickcalcs/index.cfm

## References

[1] Sllamniku S Bytyqi C Murtezani A Haxhija EQ. Correlation between avascular necrosis and the presence of the ossific nucleus when treating developmental dysplasia of the hip. *J Child Orthop.* (2013) 7:501–5. 10.1007/s11832-013-0538-z 24432113 PMC3886353

[2] ASK. *Statistikat-Afatshkurtra-te-Sherbimeve.* Kosovo: Kosovo-Agency-of-Statistics (2021).

[3] Bradley CS Perry DC Wedge JH Murnaghan ML Kelley SP. Avascular necrosis following closed reduction for treatment of developmental dysplasia of the hip: a systematic review. *J Child Orthop.* (2016) 10:627–32. 10.1007/s11832-016-0776-y 27812914 PMC5145826

[4] Novais EN Hill MK Carry PM Heyn PC. Is age or surgical approach associated with osteonecrosis in patients with developmental dysplasia of the hip? A meta-analysis. *Clin Orthop Relat Res.* (2016) 474:1166–77. 26472583 10.1007/s11999-015-4590-5 PMC4814411

[5] Ashoor M Abdulla N Elgabaly EA Aldlyami E Alshryda S. Evidence based treatment for developmental dysplasia of the hip in children under 6 months of age. Systematic review and exploratory analysis. *Surgeon.* (2021) 19:77–86. 10.1016/j.surge.2020.02.006 32249037

[6] Pavone V de Cristo C Vescio A Lucenti L Sapienza M Sessa G Dynamic and static splinting for treatment of developmental dysplasia of the hip: a systematic review. *Children (Basel).* (2021) 8:104. 10.3390/children8020104 33557053 PMC7913860

[7] Schur MD Lee C Arkader A Catalano A Choi PD. Risk factors for avascular necrosis after closed reduction for developmental dysplasia of the hip. *J Child Orthop.* (2016) 10:185–92. 10.1007/s11832-016-0743-7 27177477 PMC4909658

[8] Kalamchi A MacEwen GD. Avascular necrosis following treatment of congenital dislocation of the hip. *J Bone Joint Surg Am.* (1980) 62:876–88. 10.2106/00004623-198062060-00002 7430175

[9] Connolly P Weinstein S. The course and treatment of avascular necrosis of the femoral head in developmental dysplasia of the hip. *Acta Orthop Traumatol Turc.* (2007) 41:54–9. 17483624

[10] Niziol R Elvey M Protopapa E Roposch A. Association between the ossific nucleus and osteonecrosis in treating developmental dysplasia of the hip: updated meta-analysis. *BMC Musculoskelet. Disord.* (2017) 18:165. 10.1186/s12891-017-1468-6 28427427 PMC5397826

[11] Hernández-Cortez E. Effects of anesthesia on children’s brain development. *J Anesth Crit Care Open Access.* (2015) 2:00079.

[12] Sinem O Zeynep NO Melek C. The optimal dose of oral midazolam premedication in day case pediatric surgery. *Eurasian J Med Oncol.* (2017) 1:207–11.

[13] Bernau A. The Tübingen hip flexion splint in treatment of hip dysplasia. *Z Orthop Ihre Grenzgeb.* (1990) 128:432–5. 10.1055/s-2008-1039893 2147328

[14] Graf R. New possibilities for the diagnosis of congenital hip joint dislocation by ultrasonography. *J Pediatr Orthop.* (1983) 3:354–9. 6874934 10.1097/01241398-198307000-00015

[15] Sales de Gauzy J. Pelvic reorientation osteotomies and acetabuloplasties in children. Surgical technique. *Orthop Traumatol Surg Res.* (2010) 96:793–9. 10.1016/j.otsr.2010.07.004 20832380

[16] Walton S Schaeffer E Mulpuri K Cundy P Williams N. Evaluating the role of prereduction hip traction in the management of infants and children with developmental dysplasia of the hip (DDH): protocol for a systematic review and planned meta-analysis. *BMJ Open.* (2018) 8:e019599. 10.1136/bmjopen-2017-019599 29382681 PMC5829851

[17] Kupietzky A Houpt MI. Midazolam: a review of its use for conscious sedation of children. *Pediatr Dent.* (1993) 15:237–41. 8247896

[18] Ziesenitz VC Vaughns JD Koch G Mikus G van den Anker JN. Pharmacokinetics of fentanyl and its derivatives in children: a comprehensive review. *Clin Pharmacokinet.* (2018) 57:393–417. 10.1007/s40262-017-0609-2 28688027 PMC5756700

[19] Sasada M Smith S. *Drugs in Anaesthesia and Intensive Care.* 2nd ed. New York, NY: Oxford University Press (1997).

[20] Brennan LJ. Modern day-case anaesthesia for children. *Br J Anaesth.* (1999) 83:91–103. 10.1093/bja/83.1.91 10616337

[21] Narayanan U Mulpuri K Sankar WN Clarke NM Hosalkar H Price CT Reliability of a new radiographic classification for developmental dysplasia of the hip. *J Pediatr Orthop.* (2015) 35:478–84. 10.1097/BPO.0000000000000318 25264556 PMC4484663

[22] Bucholz RW Ogden JA. Patterns of ischemic necrosis of the proximal femur in nonoperatively treated congenital hip disease in the hip. In: Mosby CV editor. *Proceedings of the 6th Open Scientific Meeting of the Hip Society.* St Louis: C. V. Mosby Company (1978). 10.1002/ca.22245

[23] Abdallah C Hannallah R. Premedication of the child undergoing surgery. *Middle East J Anaesthesiol.* (2011) 21:165–74. 22435268

[24] Kotlarsky P Reuben H Bialik V Mark E. Developmental dysplasia of the hip: what has changed in the last 20 years? *World J Orthop.* (2015) 6:886–901. 10.5312/wjo.v6.i11.886 26716085 PMC4686436

[25] Walters JL Paule MG. Review of preclinical studies on pediatric general anesthesia-induced developmental neurotoxicity. *Neurotoxicol Teratol.* (2017) 60:2–23. 10.1016/j.ntt.2016.11.005 27871903

[26] Sun LS Li G Miller TLK Salorio C Byrne MW Bellinger DC Association between a single general anesthesia exposure before age 36 months and neurocognitive outcomes in later childhood. *JAMA.* (2016) 315:2312–20. 10.1001/jama.2016.6967 27272582 PMC5316422

[27] Soriano SG Anand KJ. Anesthetics and brain toxicity. *Curr Opin Anaesthesiol.* (2005) 18:293–7. 10.1097/01.aco.0000169238.36927.c2 16534354

[28] Warner DO Shi Y Flick RP. Anesthesia and neurodevelopment in children. *Anesthesiology.* (2018) 128:700–3. 10.1097/ALN.0000000000002121 29533967 PMC5854201

[29] Armstrong R Xu F Arora A Rasic N Syed NI. General anesthetics and cytotoxicity: possible implications for brain health. *Drug Chem Toxicol.* (2017) 40:241–9. 10.1080/01480545.2016.1188306 27252089

[30] Clarke NMP Castaneda P. Strategies to improve nonoperative childhood management. *Orthop Clin North Am.* (2012) 43:281–9. 10.1016/j.ocl.2012.05.002 22819157

[31] Sankar WN Gornitzky AL Clarke NMP Herrera-Soto JA Kelley SP Matheney T Closed reduction for developmental dysplasia of the hip. *J Pediatr Orthop.* (2019) 39:111–8. 10.1097/BPO.0000000000000895 30730414 PMC6416015

